# Consideration of communication in human–machine interaction for cooperative trajectory planning

**DOI:** 10.3389/frobt.2025.1568402

**Published:** 2025-05-06

**Authors:** Julian Schneider, Balint Varga, Sören Hohmann

**Affiliations:** Institute of Control Systems (IRS), Karlsruhe Institute of Technology (KIT), Karlsruhe, Germany

**Keywords:** communication, human–machine cooperation, shared control, cooperative trajectory planning, human–machine interaction

## Abstract

Interactive human–machine systems aim to significantly enhance performance and reduce human workload by leveraging the combined strengths of humans and automated systems. In the state of the art, human–machine cooperation (HMC) systems are modeled in various interaction layers, e.g., the decision layer, trajectory layer, and action layer. The literature usually focuses on the action layer, assuming that there is no need for a consensus at the decision or trajectory layers. Only few studies deal with the interaction at the trajectory layer. None of the previous work has systematically examined the structure of communication for interaction between humans and machines beyond the action layer. Therefore, this paper proposes a graph representation based on a multi-agent system theory for human–machine cooperation. For this purpose, a layer model for human–machine cooperation from the literature is converted into a graph representation. Using our novel graph representation, the existence of communication loops can be demonstrated, which are necessary for emancipated cooperation. In contrast, a leader–follower structure does not possess a closed loop in this graph representation. The choice of the communication loop for emancipated cooperation is ambiguous and can take place via various closed loops at higher layers of human–machine interactions, which open new possibilities for the design of emancipated cooperative control systems. In a simulation, it is shown that emancipated cooperation is possible via three variants of communication loops and that a consensus on a common trajectory is found in each case. The results indicate that taking into account cooperative strategies at the trajectory layer can enhance the performance and effectiveness of human–machine systems.

## 1 Introduction

By using human capabilities synergistically with automation, interactive human–machine systems promise to improve performance and reduce human workload, leading to a symbiotic state where human cooperation seamlessly integrates with automation (Inga et al., 2022). Such interactive human–machine systems have been studied and developed in the past in the context of driver assistance systems (Marcano et al., 2020) and teleoperation systems (Li et al., 2023; Grobbel et al., 2023) under the term shared control. Interactive wheelchair applications are another frequently considered application in this field (Carlson and Demiris, 2012). A common assumption in these works is the existence of a trajectory for the cooperative system and the knowledge of both agents about this trajectory. In the context of assisted driving, for example, this is the center of the lane as a known reference for both agents (Claussmann et al., 2020). According to Schneider et al. (2024), however, this assumption cannot generally be made. To establish a common reference between humans and automation, a cooperation process must take place at the trajectory layer (TL) in advance. For this purpose, an approach for cooperative trajectory planning is proposed in the form of an agreement process, which aims to reach a consensus on a common trajectory (Schneider et al., 2022). The application considered in this context is accompanying a patient to examination rooms.

Efficient and human-centric cooperation requires a communication channel (Lunze, 2019, p. 13). The communication between humans and automaton proposed by Abbink et al. (2018) and applied by Rothfuß et al. (2020) should take place as directly as possible and within a single layer via a separate communication interface. According to this design rule, a dedicated communication interface must be provided to agree on a common trajectory. Humans would, therefore, have to communicate their desired trajectory via an additional communication interface. However, an additional communication interface is rather unfavorable for two reasons: first, the constant communication of the desired trajectory represents an additional task for the person parallel to the execution of the movement, which can result in performance costs in the form of higher response latency or higher error rates in task execution (Fischer and Plessow, 2015). Second, according to Todorov and Jordan (2002), trajectory planning and execution in humans are often intuitive and combined, which can make it difficult for them to explicitly communicate their trajectory. Based on these limitations, the authors of this paper propose a communication method that occurs intuitively via the action layer (AL) and does not require a separate communication interface. Therefore, measured variables (state and control variables) from the action layer can be used to estimate the trajectory intentions of both agents. This approach represents an alternative communication pathway between humans and automation, differing from the approach recommended by Abbink et al. (2018).

In contrast to leader–follower approaches, an emancipated interaction should take place for the investigated cooperation at the trajectory layer as this offers advantages in the synergetic interconnection of humans and automation, e.g., in information gathering and sharing, where humans and automation can complement each other in the sensor perception of the environment in order to be able to react better to environmental influences as a result of the combination (Pacaux-Lemoine and Makoto, 2015). The underlying definition of emancipated cooperation is taken from Rothfuß (2022, p. 9), according to which humans and automation possess equal control authority.

Building on the state of the art in communication for human–machine interactions (Section 2), this paper introduces a graph-based taxonomy for describing different communication paths in Section 3. To comparatively evaluate three proposed communication paths, we conduct a simulative analysis within a cooperative positioning application of a coupled human–robot system (Section 4). Finally, Section 5 provides a summary of our key findings and concludes the work.

## 2 Related works on communication in human–machine systems

Communication is one of the central aspects of human–machine cooperation (HMC) systems. Abbink et al. (2018) established the link between shared control and communication from the Latin word “communicare,” which means “to share.” Pacaux-Lemoine and Vanderhaegen (2013) described communication between humans and automation as a necessary element of the know-how-to-cooperate (KHC) framework. This KHC, in turn, is central to keeping humans in the control loop and thus avoiding potential dangers that can arise when humans move out of it (Pacaux-Lemoine and Trentesaux, 2019). Marcano et al. (2020) stated that bidirectional communication is a necessary condition for shared control systems. Bidirectional communication is generally understood in such a way that signals can be transmitted in both directions via a communication interface. However, bidirectional communication does not necessarily have to take place via the same channel, but the signal flow only has to represent a circular structure between the sender and the receiver (Marko, 1973). The layer models of human–machine cooperation proposed by Flemisch et al. (2016), Flemisch et al. (2019), Abbink et al. (2018), and Rothfuß et al. (2020) are rather generic in character and draw general signal paths between humans and automation at all layers (Pacaux-Lemoine and Flemisch, 2019). Abbink et al. (2018) specify that communication between humans and automation should be as direct as possible and without detours, e.g., via delaying system dynamics. Losey et al. (2018) discussed different concepts of this direct haptic communication channel (kinesthetic and tactile) and the combination of channels (audio, visual, and haptic).

In the multi-agent theory, communication is also a central aspect. Lunze even stated that “cooperation needs communication” (quote from Lunze). In contrast to the classic, single-player automation design, it is not only the controller design that forms the synthesis problem, but the communication concept (which agent exchanges which information with which agent?) is also part of the synthesis problem (Lunze, 2019; Barrett and Lafortune, 1998). Individual agents that consist of controllers and corresponding actuators are modeled as nodes in a graph. When modeling a decentralized system using a graph, the immediate question that arises is which nodes are connected to each other and what type of energy and/or information is exchanged between them. The matrix that indicates which node communicates with which node is referred to as the adjacency matrix. One advantage of modeling multi-agent systems via a graph is the analysis and calculation of a consensus value, i.e., the agreement on a previously unknown common reference state (Lunze, 2019, p. 57f). Depending on the choice of the exact consensus protocol, the consensus value depends, among other things, on the initial state $x_{0}$ of the nodes. Leader–follower structures, with respect to the consensus value, are characterized in such a graph by an arrangement in which at least one node has no incoming edges, thus representing a root node of the graph (Lunze, 2019, p. 78). On the other hand, in a strongly connected node arrangement, a path can be found from each node to each other node. Each node has at least one input edge, which means that each node contributes to the formation of the consensus value.

The graph representation of multi-agent systems thus enables the description and analysis of a consensus process of an unknown reference state and the representation of different communication structures via different edge connections between nodes. This exactly fulfills the two requirements for the development of cooperation at the trajectory layer, as described in Section 1. The graph representation also enables system analysis using graph theory tools (e.g., identifying loops) and easy extensibility to integrate additional agents, e.g., when a coupled HMC system interacts with another human or agent in the environment (e.g., indicating the evasive direction to another vehicle or human or obtaining information from another robot). Therefore, a graph representation for cooperative human–machine systems will be introduced in the following section, with which three different communication paths will then be proposed in Section 4, and a simulative comparison between the communication paths will take place.

## 3 Graph-based taxonomy for the representation of communication in human–machine cooperation

From the perspective of multi-agent theory, the combination of humans and automation in the HMC context can be described as a multi-agent system consisting of two agents that communicate with each other using a respective edge configuration. In the context of haptic shared control, this is bidirectional haptic communication at the action layer via the actuator in the form of the steering wheel (Abbink and Mulder, 2010; Flad et al., 2014; Ludwig et al., 2017; Marcano et al., 2020). Communication between humans and automation can also take place symbolically via a tablet input in the case of cooperation at the decision layer (Rothfuß et al., 2020). These two examples clearly demonstrate that in HMC systems, cooperation and, thus, communication take place at different levels of task abstraction.

First, a brief clarification of the terminology used in this paper, particularly the distinction between *level* and *layer*, is necessary. As mentioned in Section 2, various layer models have been proposed in the literature for the description of HMC. These models differ in their focus mainly in three dimensions: first, the inclusion of the perception–action cycle, the degree of task abstraction, and the level of consciousness. What all models have in common is that the behavior of humans and automation is described in hierarchical levels and that these levels are mirrored across both agents. This leads to the following two definitions.

Definition 1 A **level** is a hierarchical component of the description of human or automation behavior.

Definition 2 A **layer** comprises a certain level of human behavior and the corresponding mirrored level of automation behavior together. Optionally, the layer also includes a communication interface for interactions within the layer between the two corresponding levels of the human and automation.

**FIGURE 1:**
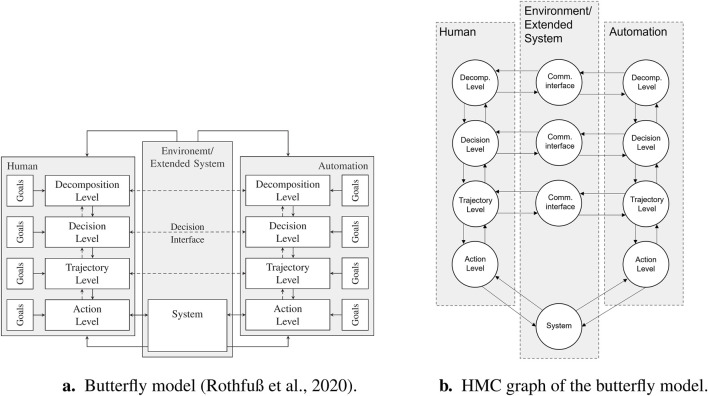
Conversion of the butterfly model (left) as a representation of an HMC model into a graph representation (right). **(a)** Butterfly model (Rothfuß et al., 2020). **(b)** HMC graph of the butterfly model.

The butterfly model presented by Rothfuß et al. (2020) is one form of an HMC layer model that focuses on the dimension of the task abstraction level. It integrates decision-making and action implementation aspects at the same time. This butterfly model was used as the underlying layer model for the present work because of its generic, application-independent character as well as its lack of restrictions with regard to authority contributions (Rothfuß, 2022, p. 26). These features make it suitable for emancipated cooperation. Taking into account Definition 1, Definition 2, the model has four layers, namely, the decomposition layer, decision layer, trajectory layer, and action layer (see Figure 1, left). In the decomposition layer, an overall task is broken down into subtasks, the sequence of which is determined in the decision layer below. In the trajectory layer, the trajectory is determined for each subtask, which is executed in the lowest layer, i.e., the action layer.

In contrast to general multi-agent systems, the two agents, humans and automation, are not each modeled as only one node for the proposed taxonomy; rather, the levels from the butterfly model are each modeled as a node (see Figure 1 right: four nodes for each agent; one node for each level). There is also a system node and a further node for each additional communication interface on the individual levels (Figure 1, center). In the general case, there are edges in both directions between all neighboring nodes. A node can only ever be connected to the node from the layer above and/or below it and to a communication interface node. Edges that run across another layer node are not permitted in the HMC graph. Figure 1 shows the general case of all possible existing nodes and edges of the graph representation of the HMC model. An instantiation of the model for a specific application generally includes only a subset of nodes and edges. An HMC graph can thus be defined as follows:

Definition 3 A **human–machine cooperation graph** G is a directed graph consisting of a node set V=H,A,S and an edge set E=P,I . The node subset H denotes all human levels contained in an HMC system. The node subset A denotes all levels of automation contained in an HMC system. The node subset S denotes all subsystems of the extended system contained in an HMC system. This includes both the system as such and all included communication interfaces. The edge subset P denotes directed physical edges between two nodes, where energy flows. The edge subset I denotes directed edges between two nodes, indicating the exchange of information between them. A directed edge from node i to node j is represented by (j→i)∈E . A node in the HMC graph G generally does not have a self-loop, i.e., an edge (j→j) .

Directed graphs can possess the property that every node can be reached from every other node, which is referred to as *strongly connected* [Lunze, p. 31]:

Definition 4 A directed HMC graph is said to be **strongly connected** if there are directed paths from a node i∈G to every other node j∈G .

### 3.1 Definition communication loop

Emancipated cooperation, as described in Section 1, is characterized by equal interaction between agents (Rothfuß, 2022, p. 9), which is the opposite of a leader–follower structure. A leader–follower structure is represented in the HMC graph as a path-like structure with a root node, i.e., one (or more) node that has only outgoing edges and no incoming edges (Lunze, 2019, p. 78). For emancipated cooperation to exist, no node should only have outgoing edges without any incoming edges. This is the case for a strongly connected graph (see Definition 4), which is a necessary condition for emancipated cooperation. Whether emancipated cooperation takes place at a layer or not can be verified in the HMC graph by identifying a loop that contains the respective level nodes of both agents.

Definition 5 **Communication loop for emancipated cooperation:** Emancipated cooperation is characterized by a closed, circular signal flow from the node of one agent to the corresponding node of the other agent in the same layer and back. In the HMC graph, this represents a cycle, i.e., a closed path from node i back to the same node i , with the condition that the path runs through the corresponding node j of the other agent in the same layer. A necessary condition for this communication loop is that the graph must be strongly connected. Depending on the application, there may be several possible communication loops.

**Remark:** the communication loop can occur either directly between two nodes via a communication interface on the same layer (intra-layer communication) or across several layers (inter-layer communication). The special case of intra-layer communication is the widespread concept of bidirectional communication, which enables the signal flow via one channel in both directions.

In the following section, the presented definition of a communication loop for emancipated cooperation is applied to three examples of possible cooperation at the trajectory layer.

### 3.2 Application of the taxonomy to cooperative approaches from the literature

**FIGURE 2:**
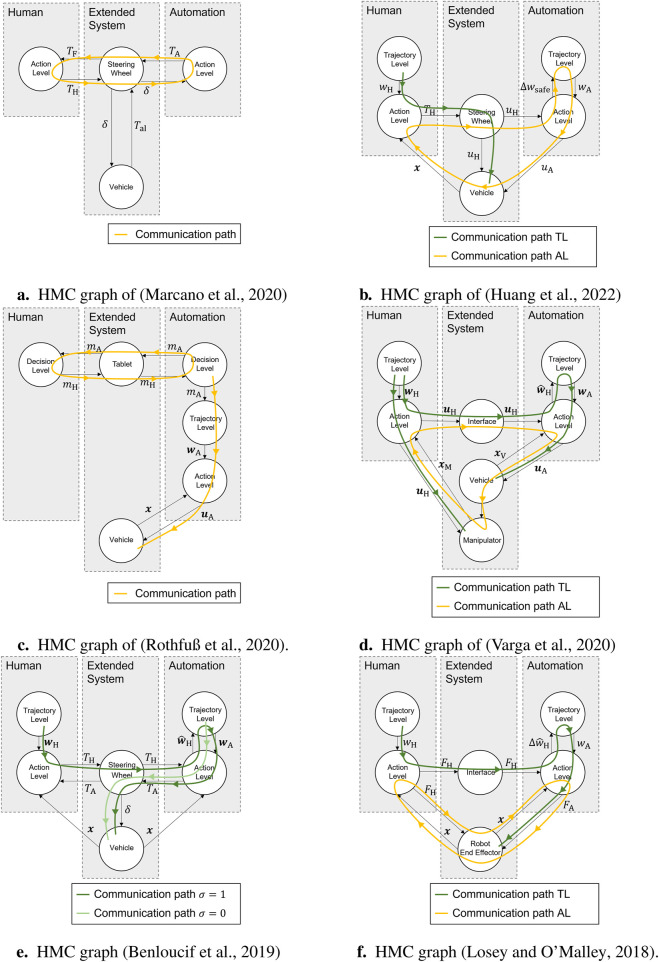
Different communication pathways in HMC graphs of cooperative approaches from the literature. **(a)** HMC graph of Marcano et al. (2020). **(b)** HMC graph of Huang et al. (2022). **(c)** HMC graph of Rothfuß et al. (2020). **(d)** HMC graph of Varga et al. (2020). **(e)** HMC graph of Benloucif et al. (2019). **(f)** HMC graph of Losey and O’Malley (2018).

Bidirectional communication plays a central role in the field of haptic shared control (Abbink et al., 2018; Marcano et al., 2020). Figure 2a shows the concept of haptic shared control in an automotive application for lateral control, represented as an HMC graph; the edge designations are taken from the haptic shared control system structure described by Marcano et al. (2020). In this case, the human and automation act simultaneously on the steering wheel as an actuator with their steering torques $T_{\text{H}}$ and $T_{\text{A}}$ , respectively. The human senses the feedback torque $T_{\text{F}}$ , and the automated system measures the resulting steering angle δ as feedback. From the steering wheel actuator, the steering angle δ acts on the vehicle dynamics. This, in turn, acts as feedback on the steering wheel actuator via the restoring torque $T_{\text{al}}$ . There is a communication loop at the action layer, with the steering wheel as the actuator representing the communication interface.

Huang et al. (2022) presented a cooperative human–machine-RRT approach that uses a safety assessment and classification of the human driving style to carry out trajectory planning, which is intended to guide the vehicle back into a defined safe space in the event of a detected unsafe driving style of the human. In the case of a detected unsafe driving style, it provides a corrective control input $u_{\text{A}}$ on the vehicle movement. Figure 2b shows the corresponding HMC graph for the lateral movement of the vehicle (longitudinal movement not shown, but it works analogously). Interaction with the human does not take place via a common communication interface. Nevertheless, there is a closed communication loop via the action layer as the control variable $u_{\text{H}}$ of the human is evaluated in order to determine the error to the safe space—noted here as $\Delta w_{\text{safe}}$ —and generate a corrective control input $u_{\text{A}}$ from this (loop shown in yellow). The human’s trajectory request $w_{\text{H}}$ is not taken into account, which means that there is no communication and, thus, no cooperation at the trajectory layer (TL) between the human and automation. The communication of the human’s trajectory shown in green is, therefore, only a path from the trajectory node to the system node. In contrast, the graph shows cooperation at the action layer, where the communication loop is closed via the system node. It has the special feature that the determination of the corrective control signal $u_{\text{A}}$ includes trajectory planning. Based on the leader–follower structure at the trajectory layer, with the human as the leader (green path), and the determination of the corrective control signal $u_{\text{A}}$ using a defined safe space, a control conflict is assumed during the phases of automation intervention.

Rothfuß et al. (2020) developed a model that describes emancipated cooperation between humans and automation at the decision-making layer in an automotive application. It examines the interaction between a human and an automated system, where both must agree on a common turning direction at a road junction (maneuver m∈{right,straight,left} , Figure 2c). The interaction takes place via a tablet. Once an agreement has been reached, the vehicle plans the trajectory $w_{\text{A}}$ of the agreed maneuver to be driven fully autonomously and also executes it fully autonomously. Figure 2c shows the HMC graph. In this case, the communication path is a loop at the decision layer, with the tablet as the communication interface.

Varga et al. (2020) presented a cooperative approach for vehicle-manipulators, in which the vehicle (automation) and the human (as the manipulator operator) cooperate. The special feature of the system dynamics is the unidirectional coupling between the vehicle and manipulator nodes (Figure 2d). The presented limited-information shared control approach results in a communication loop at the action layer, which is closed via the system of vehicle and manipulator (yellow path). The human specifies the trajectory for the manipulator (left green path). Automation estimates its reference ${\hat{w}}_{\text{A}}$ based on the human’s control variable. Both communication paths at the trajectory layer are not closed, resulting in a leader–follower structure with the human as the leader.

Benloucif et al. (2019) presented an approach that either takes into account the reference of the human (in the case of σ=1 , σ represents the driver attention state) or does not take it into account (σ=0) based on the measurement of a defined driver attention state σ∈[0,1] (Figure 2e). For 1>σ>0 , an additive compromise is formed from both references. At the trajectory layer, this results in a leader–follower structure for σ=1 and σ=0 . A compromise is found for 1>σ>0 , but this is controlled by the automation (Schneider et al., 2024).

Losey and O’Malley (2018) presented a trajectory deformation approach, which, in addition to impedance control for the movement of a robot-end effector (the yellow communication loop in Figure 2f), enables humans to deform the reference $w_{\text{A}}$ of the automation. If a force $F_{\text{H}}$ is measured, this leads to a deformation, which is referred to as $\Delta w_{\text{H}}$ in Figure 2f. The roof indicates that this is an estimated value. If the human exerts a force $F_{\text{H}}$ and is, therefore, $\Delta {\hat{w}}_{\text{H}}\neq 0$ , the human is the leader. If $\Delta {\hat{w}}_{\text{H}}=0$ , automation is the leader.

Based on the HMC graphs shown in Figure 2, it can be observed that communication paths are found in all works, which is a characteristic of cooperation (Lunze, 2019, p. 13). The most common form of cooperation occurs at the action layer (Marcano et al., 2020; Varga et al., 2020; Losey and O’Malley, 2018), with a special feature in Huang et al. (2022), where cooperation at the action layer includes the trajectory planning layer of automation. Rothfuß et al. (2020) is the only work to present a cooperative approach at the decision layer. Intra-layer communication, as defined in Section 3.1, is present in Marcano et al. (2020) and Rothfuß et al. (2020). At the trajectory layer, leader–follower structures are present. A closed communication loop at the trajectory layer is not present in any of the works presented.

The following chapter presents three different communication loops at the trajectory layer and simulates each using the same application example.

## 4 Simulative comparison of communication loops for cooperation at the trajectory layer

### 4.1 Description and motivation of the simulation system

**FIGURE 3:**
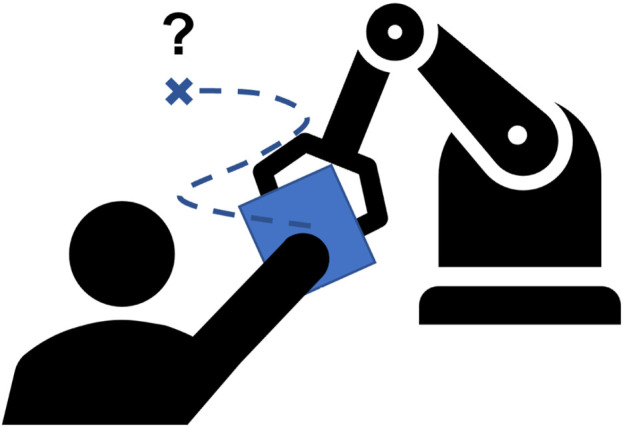
Visualization of the application example.

As an application example, a positioning task involving a heavy object is considered, performed jointly by a human and robot at the action layer. The robot is modeled as a robot arm with three degrees of freedom, which can manipulate its end effector’s position in three Cartesian dimensions: a , b , and c ^1^. For this simulation, the orientation of the end effector is neglected. Furthermore, the c -component of the positioning task is neglected, reducing the task to a planar problem in the dimensions a and b . It is assumed that the robot carries the heavy object and compensates for the weight force. The human and robot are coupled via the end effector of the robot arm (see visualization in Figure 3). The robot is operated by means of admittance control, i.e., the human can move the robot arm by exerting a two-dimensional force $F_{\text{H}}={\left[F_{\text{H},1}\;F_{\text{H},2}\right]}^{T}$ . The admittance $G_{\text{A}}$ of the robot is preset, but it is not analyzed further in this study. The robot can also adjust the position of the end effector by applying a two-dimensional force $F_{\text{A}}={\left[F_{\text{A},1}\;F_{\text{A},2}\right]}^{T}$ .

### 4.2 System model

The system dynamics result in a double integrator system of a mass-damper system, in which the force applied by the human $F_{\text{H}}$ and that applied by the robot $F_{\text{A}}$ add up and serve as input variables to the system. The state vector x consists of four state variables, defined as $x={\left[a\;\dot{a}\;b\;\dot{b}\right]}^{T}$ , and the system dynamics are described by the state-space model

$$\dot{x}=\underset{A}{\underbrace{\left[\begin{array}{cccc} 0 & 1 & 0 & 0\\ 0 & -\frac{k_{d}}{m} & 0 & 0\\ 0 & 1 & 0 & 0\\ 0 & -\frac{k_{d}}{m} & 0 & 0 \end{array}\right]}}x+\underset{B_{\text{H}}}{\underbrace{\left[\begin{array}{cc} 0 & 0\\ 0 & \frac{1}{m}\\ 0 & 0\\ 0 & \frac{1}{m} \end{array}\right]}}F_{\text{H}}+\underset{B_{\text{A}}}{\underbrace{\left[\begin{array}{cc} 0 & 0\\ 0 & \frac{1}{m}\\ 0 & 0\\ 0 & \frac{1}{m} \end{array}\right]}}F_{\text{A}},$$ (1)

$$y=\underset{C}{\underbrace{\left[\begin{array}{cccc} 1 & 0 & 0 & 0\\ 0 & 0 & 1 & 0 \end{array}\right]}}x.$$ (2)

The matrix A shows integrating behavior in both the dimensions a and b . The term $-\frac{k_{d}}{m}$ denotes the damping behavior of oscillations. For the matrices $B_{\text{H}}$ and $B_{\text{A}}$ , it is assumed that $B_{\text{H}}=B_{\text{A}}$ , which models the same force effect on the system for both the human and the robot. The two position states $x_{1}$ and $x_{3}$ result in the output y . The mass of the object to be moved is m=100 kg , and the initial state is $x_{0}={\left[2\;0\;4\;0\right]}^{T}$ .

### 4.3 Cooperation on the action layer

The interaction of the human and the robot on the action layer is modeled as a cooperative process, according to Flad et al. (2014) and Varga (2024), and it is described and solved using game theory. In this model, the human and the robot are each modeled as predictive MPCs (Flad et al., 2014), whose optimization problems are coupled. For the simulation, it is assumed that Q and R matrices are mutually known to both agents. In a practical application, these could be determined using the method described by Inga et al. (2017). In addition, both agents know the control value of the partner, which can be justified via the end effector as a haptic interface: both agents feel or measure the force of the other agent on the end effector at all times. However, the output state references $w_{\text{H}}\in R^{2}$ and $w_{\text{A}}\in R^{2}$ of both agents are not known to the respective partner and differ. With $\Delta x_{\text{H}}=x-x_{\text{H,ref}}$ , $\Delta x_{\text{A}}=x-x_{\text{R,ref}}$ , and $x_{i,\text{ref}}=\left[w_{i,1}\;0\;w_{i,2}\;0\right]$ , the cost functions of the two agents for the prediction horizon with N time steps are, therefore, as follows:

$$J_{\text{H}}=\overset{N}{\underset{k=1}{\sum }}\Delta x_{{\text{H}}_{k}}^{T}\;Q_{\text{H}}\;\Delta x_{{\text{H}}_{k}}+u_{{\text{H}}_{k}}^{T}\;R_{\text{H}}\;u_{{\text{H}}_{k}}+u_{{\text{A}}_{k}}^{T}\;R_{\text{A}}\;u_{{\text{A}}_{k}},$$ (3)

$$J_{\text{A}}=\overset{N}{\underset{k=1}{\sum }}\Delta x_{{\text{A}}_{k}}^{T}\;Q_{\text{A}}\;\Delta x_{{\text{A}}_{k}}+u_{{\text{A}}_{k}}^{T}\;R_{\text{A}}\;u_{{\text{A}}_{k}}+u_{{\text{H}}_{k}}^{T}\;R_{\text{H}}\;u_{{\text{H}}_{k}}.$$ (4)

Q and R matrices have the following entries:

$$\begin{array}{ll} Q_{\text{H}} & =\left[\begin{array}{cccc} 500 & 0 & 0 & 0\\ 0 & 0.1 & 0 & 0\\ 0 & 0 & 500 & 0\\ 0 & 0 & 0 & 0.1 \end{array}\right],\\ Q_{\text{A}} & =\left[\begin{array}{cccc} 1000 & 0 & 0 & 0\\ 0 & 0.1 & 0 & 0\\ 0 & 0 & 1000 & 0\\ 0 & 0 & 0 & 0.1 \end{array}\right],\\ R_{\text{H}} & =\left[\begin{array}{cc} 0.8 & 0\\ 0 & 0.8 \end{array}\right],\\ R_{\text{A}} & =\left[\begin{array}{cc} 0.3 & 0\\ 0 & 0.3 \end{array}\right]. \end{array}$$

It is assumed that the human penalizes deviations in state less than the automation does. At the same time, the human assigns a higher penalty to the control variable, which should lead the robot to take on a greater share of the load.

**FIGURE 4:**
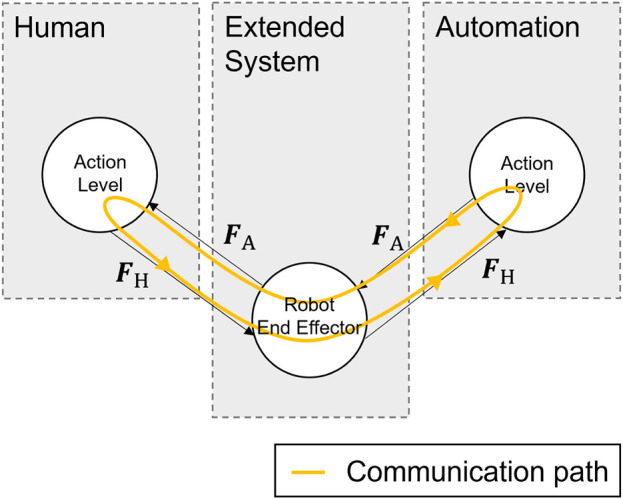
HMC graph showing the communication loop for cooperation on the action layer.

The solution for the control variables $u_{\text{H}}$ and $u_{\text{A}}$ results from the Nash equilibrium. The solutions of the coupled MPC optimization problems are calculated using the iterative best response method, according to which each agent solves its own coupled MPC optimization problem and that of its partner at each time step. In one iteration, the control variable of the partner is kept constant, and its own control variable is calculated. In the next iteration, this control value is kept constant for the partner’s optimization problem, and the partner’s control variable is updated. This update process continues until the control values converge to the Nash equilibrium. The optimization problems were solved in this paper using CasADi (Andersson et al., 2019) and an IPOPT solver. According to the receding horizon strategy, only the first element of the control variable trajectory is fed into the system (Kwon and Han, 2005). The described modeling of the action layer can be depicted using the graph shown in Figure 4. forces $F_{\text{H}}$ and $F_{\text{A}}$ serve as the communication variables.

**FIGURE 5:**
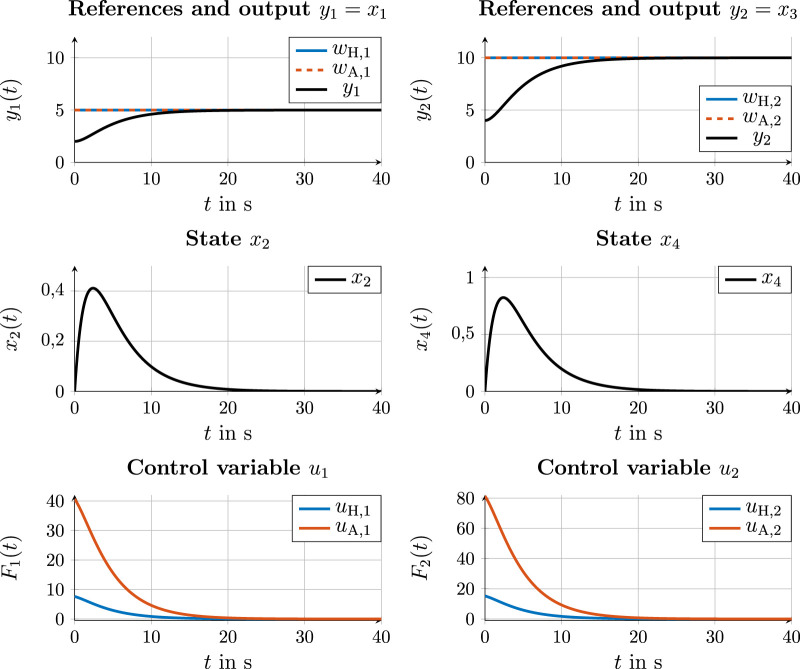
Simulation results for state and control values on the action layer with human and robot having the same reference $w_{\text{H}}=w_{\text{A}}$ for the output states.

Figure 5 shows a simulation in which $w_{\text{H}}$ and $w_{\text{A}}$ initially match: $w_{H}=w_{A}={\left[5\;0\;10\;0\right]}^{T}$ . In the state progression, it can be observed that the states $x_{1}$ and $x_{3}$ converge to the references $w_{1}$ and $w_{3}$ in the steady state, respectively. In addition, it can be observed in the control variable curve that both control variables $u_{\text{H}}$ and $u_{\text{A}}$ disappear for t→∞ .

**FIGURE 6:**
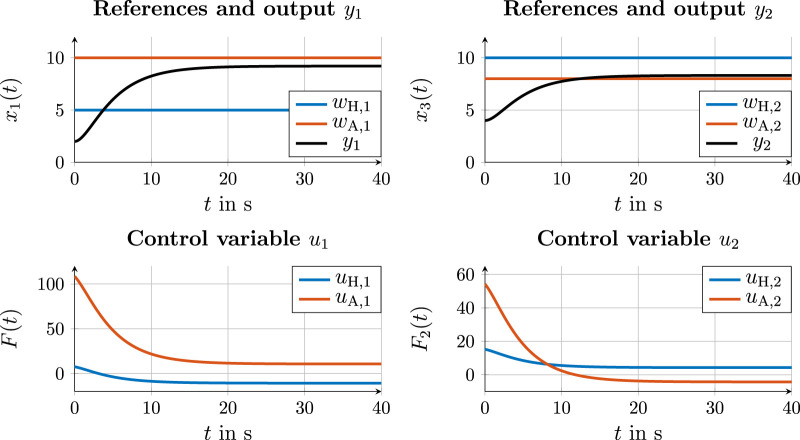
Simulation results for state and control values on the action layer with the human and robot having different references.

Figure 6, on the other hand, shows a simulation in which $w_{\text{H}}$ and $w_{\text{A}}$ differ ( $w_{\text{H}}={\left[5\;10\right]}^{T}$ and $w_{\text{A}}={\left[10\;8\right]}^{T}$ ), and it can be seen that for the outputs $y_{1}$ and $y_{2}$ , there is a constant steady state value for t→∞ . However, this constant steady state value lies between the two references of the human and the robot. It can be observed that it is closer to the reference $w_{\text{A}}$ in each case. This can be explained by the higher control values of the robot during 0 s<t<8 s , which are also shown in the diagram in Figure 6. The higher control values of the robot result from the lower penalty of the control variable in the $R_{\text{A}}$ matrix compared to the $R_{\text{H}}$ matrix (see (4)), whereby higher control values are permitted for the robot. In this simulation, this corresponds to the intention that the robot takes on a higher load of the weight to be moved than the human. When examining the control values $u_{\text{H},1}$ and $u_{\text{A},1}$ as well as $u_{\text{H},2}$ and $u_{\text{A},2}$ , it is noticeable that these do not disappear in the steady state for t→∞ ; instead, they settle atthe following values: $u_{\text{H},1}\approx 10.7\text{ N}$ and $u_{\text{A},1}\approx 10.7\text{ N}$ and $u_{\text{H},2}\approx 4.3\text{ N}$ and $u_{\text{A},2}\approx \text{−}4.3\text{ N}$ . This is the control conflict between the robot and the human described in Section 1. This arises from the Nash equilibrium of the interaction at the action layer, which results in constant steady-state values $y_{1}$ and $y_{2}$ . However, these constant steady-state values are based on the force equilibrium $u_{\text{H}}=-u_{\text{A}}$ between the human and the robot. This force equilibrium can be explained by the fact that $\Delta x_{\text{H}}$ and $\Delta x_{\text{A}}$ do not become 0, whereby, according to (3) and (4), a non-vanishing control value results for both agents. This control conflict is to be resolved by reaching a consensus on a common reference so that the control error e disappears for t→∞ . This requires cooperation at the trajectory layer, which is introduced and simulated in the following subsection.

### 4.4 Cooperation on the trajectory layer

**FIGURE 7:**
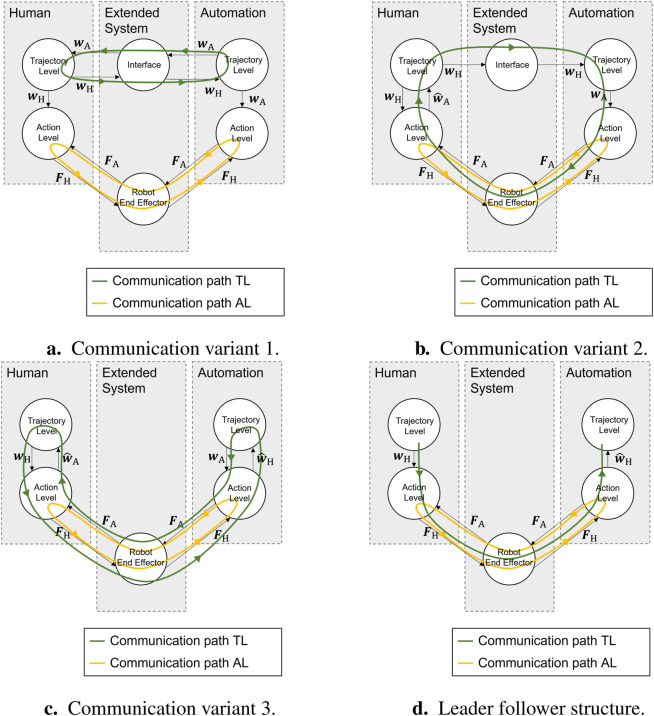
Communication variants 1, 2, and 3 for emancipated cooperation and a leader–follower structure in (d). **(a)** Communication variant 1. **(b)** Communication variant 2. **(c)** Communication variant 3. **(d)** Leader–follower structure.

To achieve consensus at the trajectory layer, the graph from Figure 4 must be extended to the trajectory layer. From a development perspective, there are several options for selecting the communication loop, as required in Definition 5. Three such options are shown in Figure 7, which are compared in the following subsections. In addition, a leader–follower simulation is carried out for comparison (see Figure 7d).

In all cases, a simple consensus protocol from Lunze (2019) (p. 58) is used, whose model dynamics are integrating and take the estimated error $\Delta \hat{w}=w_{\text{i}}-w_{\text{j}}$ as input. This consensus protocol can deviate from real human behavior; however, it is suitable for demonstrating the working principle of the proposed communication loops. For a practical application, a human consensus protocol would first have to be identified in a study. The consensus protocol at the trajectory layer for an agent in the present case is defined as follows:

$${\dot{w}}_{i}=-a_{ij}\left(w_{i}\left(t\right)-{\hat{w}}_{j}\left(t\right)\right),\;i,j\in \left\{\text{H},\text{A}\right\}.$$ (5)

For both agents, $a_{ij}=0.12$ was chosen. The reference values of the human and the robot for the following simulations are as follows:

$$w_{\text{H}}={\left[5\;10\right]}^{T},$$ (6)

$$w_{\text{A}}={\left[10\;8\right]}^{T}.$$ (7)

In both dimensions of the reference vectors, there is, therefore, a disagreement between the human and the robot. Due to the symmetry of the inertia in both dimensions of the heavy object to be moved and the fact that the two dimensions are not coupled, only the first output $y_{1}$ is considered below for reasons of space. The results for the second output $y_{2}$ are analogous to the output $y_{1}$ .

#### 4.4.1 Communication variant 1

**FIGURE 8:**
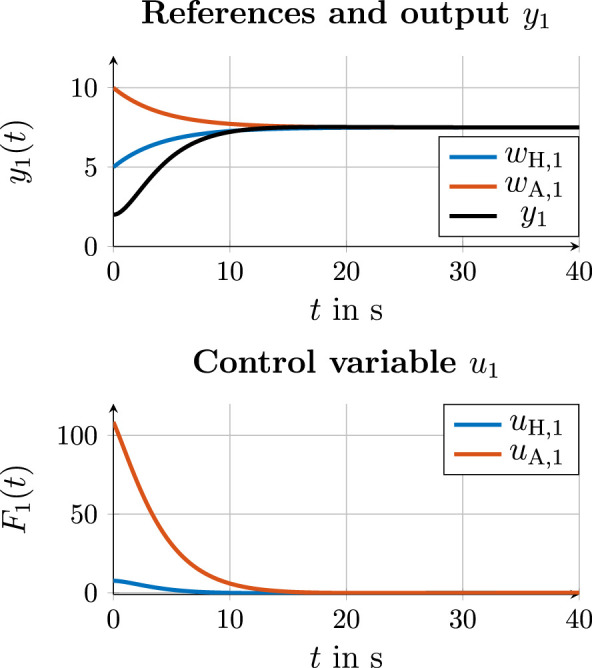
Simulation results for state and control values with cooperation on the trajectory layer in communication variant 1.

As the first option, the communication variant proposed by Abbink et al. (2018) is simulated, in which a separate communication interface is established at the trajectory layer. In practice, this can be achieved via a tablet or a second joystick. For this simulation example, it is assumed that the human and the robot communicate their references $w_{\text{H}}$ and $w_{\text{A}}$ directly and explicitly via an unspecified interface. The estimated value ${\hat{w}}_{j}\left(t\right)$ in the consensus protocol (5) thus becomes the true value $w_{j}\left(t\right)$ . Figure 8 shows the output $y_{1}$ and the consensus for the references $w_{\text{H},1}$ and $w_{\text{A},1}$ . The selected consensus protocol (5) leads, according to equation (3.18) in Lunze (2019), p. 66),

$$\bar{w}=\frac{1}{N}\overset{N}{\underset{i=1}{\sum }}w_{i},$$ (8)

to a consensus with the value $\bar{w}=7.5$ with N=2 , $w_{\text{H}}=5$ , and $w_{\text{A}}=10$ . It can be observed that at the action layer, the output $y_{1}$ takes the found consensus in the reference value as its steady-state value. This, in turn, means that the control values disappear as desired in the steady-state case (see Figure 8).

#### 4.4.2 Communication variant 2

The second communication variant is based on the communication loop presented by Schneider et al. (2022), as shown in Figure 7b for this simulation example. Humans communicate their reference to the robot via an interface. The automation in turn responds to its reference request by executing a movement, i.e., via the action layer. Reasons for choosing this communication loop can be safety aspects, as is the case described by Schneider et al. (2022): for safety reasons, the robot arm, as the communication interface from automation to the human, must not be moved. The reference value ${\hat{w}}_{A}$ of the automation has to be reconstructed from the control variables of automation at the action level. For this purpose, a heuristic was applied that controls the error $\Delta u_{\text{A}}$ to 0 . This control error is the difference between the measured control variable of automation $u_{\text{A}}$ and a control variable ${\hat{u}}_{\text{A}}$ calculated using the known $Q_{\text{A}}$ and $R_{\text{A}}$ matrices of automation with an estimated reference ${\hat{w}}_{\text{A}}$ . The error

$$\Delta u_{\text{A}}=u_{\text{A}}-{\hat{u}}_{\text{A}}$$ (9)

is added to the current estimated value ${\hat{w}}_{\text{A}}$ at each time step after multiplication with a constant gain $P_{u_{\text{A}}}$ as follows:

$${\hat{w}}_{\text{A}}\left(t=\left(k+1\right)T\right)={\hat{w}}_{\text{A}}\left(t=kT\right)+P_{u_{\text{A}}}\Delta u_{\text{A}}.$$ (10)

With the new estimated value ${\hat{w}}_{\text{A}}\left(t=\left(k+1\right)T\right)$ , a new input ${\hat{u}}_{\text{A}}\left(t=\left(k+1\right)T\right)$ is calculated in the forward simulation via the MPC solution of automation, and this allows the new control error $\Delta u_{\text{A}}$ to be measured. The parameter $P_{u_{\text{A}}}$ is chosen as

$$P_{u_{\text{A}}}=\left[\begin{array}{cc} 0.05 & 0\\ 0 & 0.05 \end{array}\right].$$ (11)

**FIGURE 9:**
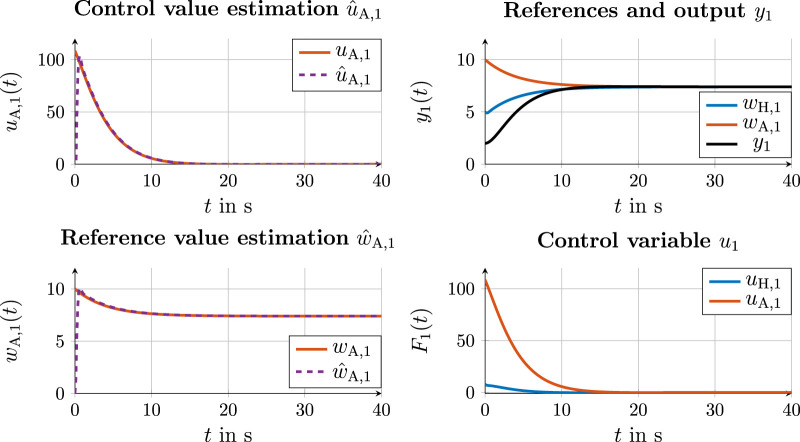
Simulation results for the control value and state estimation of automation (left) and state and control values with cooperation on the trajectory layer in communication variant 2 (right).

Figure 9 shows (the two diagrams on the left) the corresponding resulting curves for the estimation of ${\hat{u}}_{\text{A},1}$ and ${\hat{w}}_{\text{A},1}$ . It shows that the estimate ${\hat{u}}_{\text{A},1}$ after t≈8 s corresponds approximately to the true manipulated variable $u_{\text{A},1}$ . The estimate of the reference ${\hat{w}}_{\text{A},1}$ corresponds to the true reference $w_{\text{A},1}$ after t≈10 s . The estimation of ${\hat{w}}_{\text{A},1}$ results, according to the consensus Formula 5, in the reference curves for $w_{\text{H},1}$ and $w_{\text{A},1}$ , as shown in the right top of Figure 7b. The resulting control variable curves $u_{\text{H},1}$ and $u_{\text{A},1}$ are shown on the bottom right. It can be observed that the control variables for t→∞ disappear as desired, and the output $y_{1}$ converges to the steady-state value, which corresponds to the found reference consensus.

#### 4.4.3 Communication variant 3

The third alternative is the communication shown in Figure 7c. Compared to communication variant 2, humans also communicate their desire to move via the direct execution of their movement. Both agents use the variables from the underlying action layer as input variables for cooperation at the trajectory layer, and each agent reconstructs the reference variables of the partner from the feedback of the error $\Delta u_{\text{A}}$ = $u_{\text{A}}-{\hat{u}}_{\text{A}}$ and $\Delta u_{\text{H}}$ = $u_{\text{H}}-{\hat{u}}_{\text{H}}$ , respectively. The automation also estimates the reference of the human according to (Equation 10). Here, the control error $\Delta u_{\text{H}}$ is multiplied with the gain.

$$P_{u_{\text{H}}}=\left[\begin{array}{cc} 0.01 & 0\\ 0 & 0.01 \end{array}\right].$$ (12)

**FIGURE 10:**
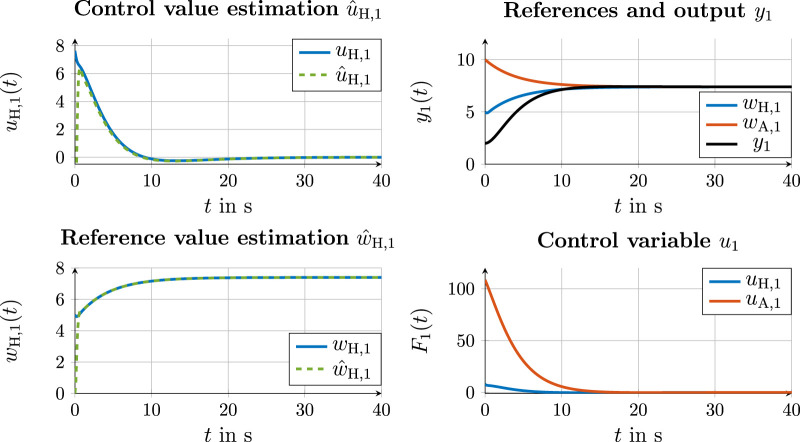
Simulation results for the control value and state estimation of automation (left) and state and control values with cooperation on the trajectory layer in communication variant 3 (right).

The estimated values for ${\hat{u}}_{\text{H},1}$ and ${\hat{w}}_{\text{A},1}$ are shown in the two diagrams in the left in Figure 10. After t≈10 s , the estimated control value follows its true value. For reference, the estimation closely follows the true value after t≈4 s . Using these estimated reference values, the consensus at the trajectory layer, along with the resulting state and control values, is shown on the right side of Figure 10.

#### 4.4.4 Leader–follower communication

**FIGURE 11:**
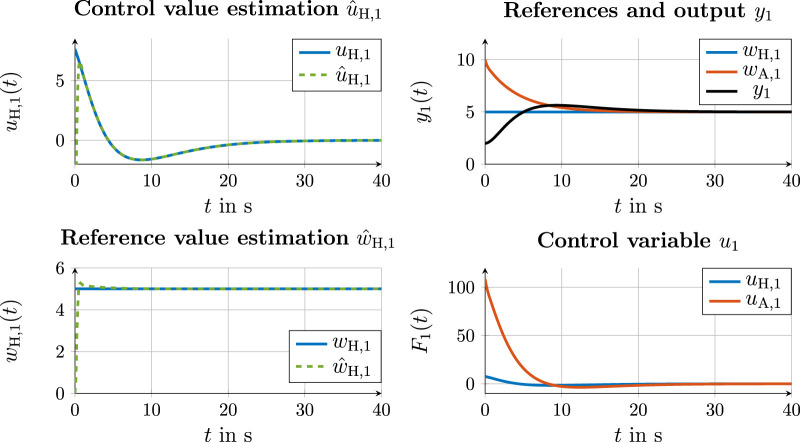
Simulation results for the control value and state estimation of the human (left) and state and control values (right) with leader–follower communication on the trajectory layer.

Compared to emancipated cooperation approaches and associated communication variants 1–3, the graph in Figure 7d shows a leader–follower structure in which the human is the leader for the reference. The communication path from the human to automation corresponds to that of communication variant 3, in which automation estimates the desired reference of the human from the state and control variables of the action layer. Figure 11 shows the estimation curves for ${\hat{u}}_{\text{H},1}$ and ${\hat{w}}_{\text{H},1}$ . Based on the estimated reference ${\hat{w}}_{\text{H},1}$ of the human, the consensus protocol for consensus finding at the trajectory layer with $a_{\text{HA}}=0.25$ results in the two trajectories for $w_{\text{H,}1}$ and $w_{\text{A,}1}$ , as shown in Figure 11 (right). The reference of automation matches the reference of the human as the leader until they align with each other after t≈20 s . The output $y_{1}$ follows the human reference and is adjusted after t≈25 s .

**FIGURE 12:**
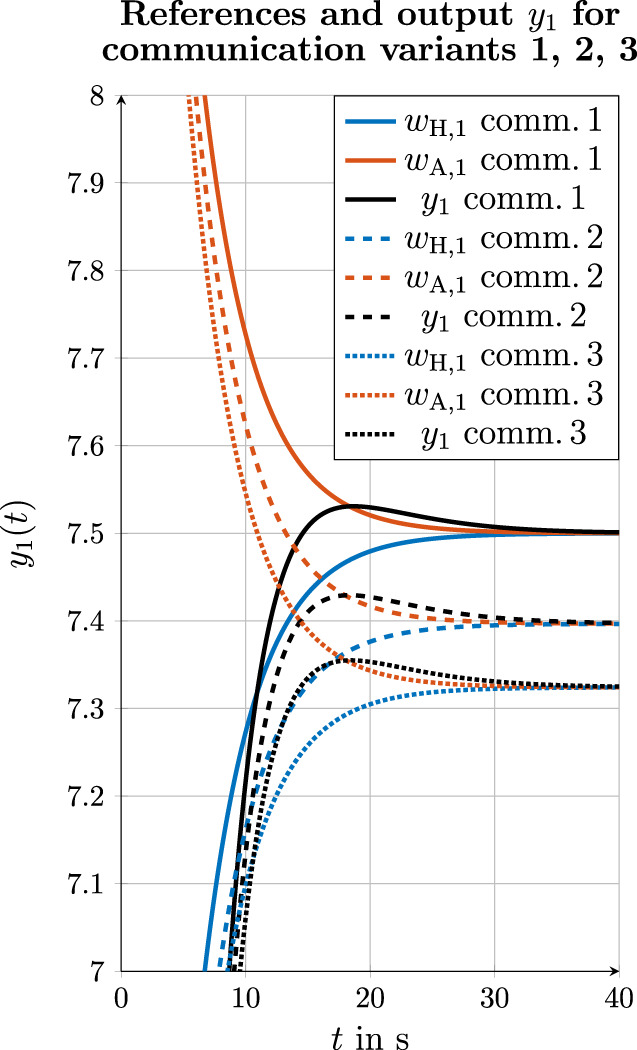
Simulation results for references and output $y_{1}$ for all three communication variants.

### 4.5 Comparison of the consensus values in communication variants 1, 2, and 3

As it can be observed in the simulation results in Sections 4.4.1, Section 4.4.2, and Section 4.4.3, a consensus between the human and automation is found in all three communication variants using the consensus protocol (5). According to Equation 3.18 from Lunze (2019), the consensus $\bar{w}=7.5$ exists for the references for output $y_{1}$ in the case of the exchange of the true reference values $w_{\text{H}}$ and $w_{\text{A}}$ . However, the estimation ${\hat{w}}_{i}$ from the control variables $u_{i}$ generates an estimation error at the beginning of cooperation for small t :

$$e_{\text{estim},j}=w_{j}-{\hat{w}}_{j}.$$ (13)

As a result, the estimation error (Equation 13) applies to the consensus protocol:

$${\dot{w}}_{i}=-a_{ij}\left(w_{i}\left(t\right)-{\hat{w}}_{j}\left(t\right)\right),\;i,j\in \left\{\text{H},\text{A}\right\},$$ (14)

$${\dot{w}}_{i}=-a_{ij}\left(w_{i}\left(t\right)-\left(w_{j}\left(t\right)-e_{\text{estim},j}\left(t\right)\right)\right).$$ (15)

As shown in Figures 9, 10 for the estimation of references, the estimation error $e_{\text{estim},j}$ disappears for t→∞ . However, in the comparison of consensus values of all three communication variants, a difference can be observed according to the existent estimation error $e_{\text{estim},j}$ . Figure 12 shows the reference $w_{\text{H},1}$ of the human, the reference $w_{\text{A},1}$ of the automation, and the output $y_{1}$ for all three communication variants. It can be observed that in the first communication variant, in which the true references $w_{\text{H},1}$ and $w_{\text{A},1}$ are exchanged, the consensus ${\bar{w}}_{\text{comm. }1}=7.5\text{ m}$ is reached according to (Equation 8). In the second communication variant, ${\bar{w}}_{\text{comm. }2}=7.40\text{ m}$ is reached, and in the third communication variant, ${\bar{w}}_{\text{comm. }3}=7.32\text{ m}$ is reached. Compared to the first communication variant, the third communication variant, therefore, shows a difference of 0.18 m to ${\bar{w}}_{\text{comm. }1}$ . At the same time, it must be said that this difference is the result of the present simulation of the human behavior. In practice, it is expected that humans will continue to apply a control variable $|u_{\text{H}}|>0$ if they have not yet agreed with the consensus reached with automation.

### 4.6 Discussion

The simulation results show that for emancipated cooperation at the trajectory layer, a consensus for the common reference is found in all three cases shown, each involving a variation of the communication loops. It should be noted that the selected consensus protocol (Equation 5) from Lunze (2019) represents a model that does not always align with human behavior. In practice, this consensus behavior of humans must first be identified since it may vary between human groups, e.g., concerning age and gender. Second, for the first communication variant in Section 4.4.1, a perfect exchange of information about the true references between the two agents was assumed. In practice, however, this always happens via some type of communication interface. Depending on the communication medium used and the ability of humans to communicate their true reference via this, an error in the true reference is expected in a practical setup. Finally, it was assumed for all simulations that all necessary parameters are known, particularly the entries of the Q and R matrices, are known to both agents. These parameters would need to be identified first in practical use. Despite these limitations and simplifications mentioned, the simulations provide a successful proof of concept for variants of communication loops for emancipated cooperation at the trajectory layer.

## 5 Conclusion

In this article, a graph representation for a human–machine cooperation system was presented. For this purpose, the butterfly layer model from Rothfuß et al. (2020) was converted into a graph representation. The graph representation offers several advantages: on one hand, human–machine cooperation systems can easily be extended to cases involving interactions with other agents. On the other hand, the graph can be examined for closed-loop communication between the human and automation by checking for loops in the form according to Definition 5. For practical application, a human consensus protocol must first be identified through a study. In addition, the next step is to conduct a study to test the proposed communication loops with humans in practice.

## Data Availability

The raw data supporting the conclusions of this article will be made available by the authors, without undue reservation.

## Footnotes

1 The denotations a , b , and c are used instead of x , y , and z for the three spatial directions in order to avoid confusion with the system states x and system outputs y .
